# Correspondence

**Published:** 1881-10

**Authors:** 


### CORRESPONDENCE.

### CINCINNATI LETTER—THE AMERICAN ASSOCIATI N FOR THE ADVANCEMENT OF SCIENCE.

To return to the meeting of the Association for the Advancement of Science,we repair to Music Hall. The grand vestibule is filled with people of both sexes and of the most diverse appearances. Fine looking spectacled and sleek-headed old men, long-haired hollow-eyed and lean individuals, long and dusty-coated persons with a look of rural simplicity about them, smart and delicate looking men, portly old ladies, and spinsters of many types are passing and repassing or conversing together in groups. Along the corridors and stairways are people hurrying to and fro to attend the meetings of the sections and sub-sections, while the local committee are at work preparing the daily lunch. The immense buildings are cobwebbed with telephone wires, so that the sections can communicate instantaneously, and diagrams are placed in each room to indicate the progress of the reading of papers.

This from day to day has been the scene at Music Hall, and though the sub-sections were not always crowded the general attendance was good, and the patronage of the lunches excellent.

It would be entirely out of the way for me to attempt to follow the long and diverse series of papers which were brought before this association. I will only mention those which would be likely to interest the medical man.

Among the most interesting was the paper of Dr. Harrison Allen, of Philadelphia, on the ethmoid bone. It was illustrated with specimens and blackboard drawings, and created considerable interest. The author has studied the comparative development of this bone in quite a number of animals, and has discovered a regularity in the development of the ethmoidal plates corresponding to the acuteness of the sense of smell. In confirmation of this point he exhibited the bone from the dog in contradistinction to that of man, the former having several of the so-called ecto-turbinated processes while the latter has none. The point that Dr. Allen made can be fully appreciated when we remember how infinitely superior in development the sense of smell in the former is to that in the latter.

The paper of Dr. Geo. M. Sternberg of the United States Army, discussed the life-history and nature of the parasites which infest the mucous surfaces of the body, especially those of the mouth and urethra, and was mumerously illustrated with micro-photographs. According to him when healthy urine becomes alkaline it undergoes the change in consequence of the introduction of germs from without generally by the use of a filthy instrument, as they could penetrate but a short distance beyond the meatus without such aid. From this it may be inferred that ordinary cases of alkaline urine show disease of the kidneys.

Dr. Frank W. Langdon, of Cincinnati, read a paper on "The Temporal process of the malar bone in the ancient human crania from Madisonville. This paper was devoted to a hitherto undescribed process on the posterior border of the malar bone, which he said is present in more than nine-tenths of these ancient skulls.

Judge Henderson gave a lecture on "Ilex Cassine, the black drink of the Southern Indians;" and handed around cups of the drink, steaming hot, to the audience.

Dr. Lester Curtis, of Chicago, read a paper on "The study of the blood after a protracted fast," based on the recent forty-five days' fast of John Griscom, at Chicago. The fast, he said, was a genuine one. The blood was examined microscopically immediately after the last meal and on each succeeding day of the fast. After the second day the corpuscles began to show abnormal appearances changing form almost every day. Diagrams were shown exhibiting them as serrated, pear shaped, or drawn out at considerable length.

Dr. D. W. Prentiss, of Washington, D. C., read a very interesting paper on "The Effects of Pilocarpin on the Growth and Color of the Hair." His observations were founded mainly on a case of uræmia, where the drug was administered with entirely different expectations. In enumerating the effects of the drug upon the system, he speaks as follows: "The drug was administered by hypodermic injection, in the dose of one centigram (gr. 1-6). Immediately, almost before the needle was withdrawn, the face and neck would flush up to a bright red, and dimness of vision be noticed. This was shortly followed by palpitation. In three minutes there was slight nausea, with the eye, nose and mouth beginning to water, and the skin showing moisture. In seven minutes there was free vomiting, profuse sweating and salivation. The action of the drug lasted from four to six hours. The vomiting continued throughout the period of action, almost without intermission, and was the most distressing symptom of the ordeal.

In the beginning of the sweat the water flowed freely from the eyes and nose as well as the mouth, but when the salivation became fully established, the eyes and nose ceased to discharge. The saliva was viscid and tenacious, so that to clear the mouth it was necessary to use a handkerchief. Its flow was so profuse that, after thus clearing the mouth, she (a lady twenty-five years old and weighing 100 lbs.) would not have time to get a drink of water before the mouth would again be filled. So also talking connectedly was altogether prevented. The amount of fluid discharged from the mouth, including that vomited, was never less than two quarts, and often as much as a gallon.

The perspiration was first noticed on the forehead and neck; then the skin of the whole body, which had previously been dry and harsh, became moist. When the sweating was fully established, the water ran in little streams over all the parts of the body, and in the face was with difficulty kept out of the eyes. In five minutes the hair would be perfectly saturated. * * * The odor of the perspiration was offensive, and on some occasions had an urinous smell. The pupils were contracted to a small point. The sight became impaired at the first rush of blood to the head, and the dimness of vision continued to increase until it was impossible for her to distinguish objects beyond the foot of the bed.

As the effects of the drug wore off, the exhaustion was extreme—pulse 130 and feeble. * * * It was estimated that the amount of fluid discharged during each sweat was not less than fourteen pints. This seems incredible, but is nevertheless true. * * * The period covered by the use of the drug was from Dec. 16th, 1880, to Feb. 22d, 1881. Between these dates twenty-two sweats were given, and between thirty-five and forty centigrams of pilocarpin used. The hair was first noticed to be darker on Dec. 27th (it being originally of a light blonde color). On Jan. 12th it was very dark, and in May the change to black was complete." As late as August, 1881, the hair was still very black.

In addition to the effect of darkening the hair, it was claimed that pilocarpin stimulates its nutrition, as the last specimens exhibited were markedly coarser and better nourished than the first. According to this it was believed that dark hair is generally more vigorous than that of lighter color.

Dr. H. D. Schmidt, of New Orleans, read a paper on "the influence of the structure of the nerve fibres upon the production and conduction of nerve force." I regret exceedingly my inability to give any points from the paper, not having been present at the reading and being unable to find the author. The meetings of the Association are now over, having ended in two grand complimentary excursions—one to Mammoth Cave, the other to Lookout Mountain. The late meeting is spoken of everywhere as a success; but judging from past expectations as published in several newspaper articles I should think it fell short in numbers. Of the two thousand visitors who were expected only, five hundred and thirty-one had registered up to the evening before adjournment, and about half of these were new members, the majority of whom are Cincinnatians. The finances of the association must have been materially increased, as new members with their eight dollars were accepted from every source and no questions asked.

The next meeting will be held in Montreal on the fourth Wednesday in August, 1882.

